# Application of Bayesian statistics to estimate nitrous oxide emission factors of three nitrogen fertilisers on UK grasslands

**DOI:** 10.1016/j.envint.2019.04.054

**Published:** 2019-07

**Authors:** N. Cowan, P. Levy, J. Drewer, A. Carswell, R. Shaw, I. Simmons, C. Bache, J. Marinheiro, J. Brichet, A.R. Sanchez-Rodriguez, J. Cotton, P.W. Hill, D.R. Chadwick, D.L. Jones, T.H. Misselbrook, U. Skiba

**Affiliations:** a Centre for Ecology and Hydrology, Bush Estate, Penicuik, Midlothian, UK; b Rothamsted Research, Sustainable Agriculture Sciences, North Wyke, Devon, UK; c School of Natural Sciences, Bangor University, Gwynedd, UK; d Instituto Superior de Agronomia, Universidade de Lisboa, Tapada da Ajuda, Lisboa, Portugal; e Departamento de Agronomía, Universidad de Córdoba, ETSIAM, Córdoba, Spain; f UWA School of Agriculture and Environment, University of Western Australia, Crawley, Australia

**Keywords:** Agriculture, N_2_O, Urease inhibitor, Urea, Uncertainty

## Abstract

Trapezoidal integration by linear interpolation of data points is by far the most commonly used method of cumulative flux calculations of nitrous oxide (N_2_O) in studies that use flux chambers; however, this method is incapable of providing accurate uncertainty estimates. A Bayesian approach was used to calculate N_2_O emission factors (EFs) and their associated uncertainties from flux chamber measurements made after the application of nitrogen fertilisers, in the form of ammonium nitrate (AN), urea (Ur) and urea treated with Agrotain® urease inhibitor (UI) at four grassland sites in the UK. The comparison between the cumulative fluxes estimated using the Bayesian and linear interpolation methods were broadly similar (R^2^ = 0.79); however, the Bayesian method was capable of providing realistic uncertainties when a limited number of data points is available. The study reports mean EF values (and 95% confidence intervals) of 0.60 ± 0.63, 0.29 ± 0.22 and 0.26 ± 0.17% of applied N emitted as N_2_O for the AN, Ur and UI treatments, respectively. There was no significant difference between N_2_O emissions from the Ur and UI treatments. In the case of the automatic chamber data collected at one site in this study, the data did not fit the log-normal model, implying that more complex models may be needed, particularly for measurement data with high temporal resolution.

## Highlights

• Bayesian statistics provided realistic uncertainties in cumulative N_2_O flux estimates after fertiliser application.
• No significant difference in emissions of N_2_O were observed between urea fertiliser and urea treated with urease inhibitor.
• Emissions of N_2_O after the application of ammonium nitrate fertilisers is shown to be significantly higher than those of urea based fertilisers.

## 1 Introduction

Agriculture contributes an estimated 60–70% to global anthropogenic nitrous oxide (N_2_O) emissions (Syakila and Kroeze, 2011; Tian et al., 2019), primarily due to increased application of reactive nitrogen (Nr) fertilisers to soils and subsequently aquatic systems, from which N_2_O is released as a byproduct of the microbial processes of nitrification and denitrification (Davidson et al., 2000). N_2_O is a potent greenhouse gas as well as the most significant contributor to global stratospheric ozone depletion (Ravishankara et al., 2009), which doubly increases the incentive to mitigate these emissions. Current projections predict that global rates of Nr fertilisation will continue to rise over the next century to cope with a growing population, changing diets and greater demand for food. Therefore, it has become increasingly urgent to address the issue of N_2_O emissions from agriculture sources. However, food supply is a sensitive issue both politically and economically, and there are a limited number of mitigation options available that may reduce agricultural N_2_O emissions without impacting crop yields.

Chemical inhibitors that target urease hydrolysis and microbial nitrification are commercially available and have been shown to reduce Nr losses under laboratory conditions and in field trials, but with varying success. Microbial inhibitors and compounds which block enzymes of microbially mediated pathways are also in development (Sanz-Cobena et al., 2016; Ni et al., 2014; Singh et al., 2013; Rose et al., 2017; Ruser and Schulz, 2015). Although there are many positive studies, which promote the pollution-reducing capabilities of these inhibitors, especially the reduction of NH_3_ losses, some questions remain over the overall effectiveness of the inhibitors, which face claims that reduction of NH_3_ losses may increase N_2_O emissions (Lam et al., 2016; Carswell et al., 2018).

Serious difficulties lie in the estimation of accurate emission factors from experiments investigating N_2_O fluxes, due to the measurement methods available and the unpredictable heterogeneous nature of microbial production of N_2_O (Butterbach-Bahl et al., 2013). As such, it can be difficult to accurately assess the true impact of any particular N_2_O mitigation effort. The majority of N_2_O flux studies (past and present) deploy chamber methodology to measure emissions from soils (Dobbie et al., 1999; Chadwick et al., 2014; Cowan et al., 2015). These chambers are sampled periodically, and only represent a small surface area of soil (chamber sizes typically vary from 10 cm^2^ to 1 m^2^). Due to the unpredictable spatial and temporal variability in N_2_O fluxes from agricultural soils, estimates of emissions of N_2_O after fertiliser events have large associated uncertainties (Cowan et al., 2017; Levy et al., 2017).

In the absence of reliable predictive models, trapezoidal (linear) integration between points is by far the most commonly used method of cumulative flux estimation. However, this method does not provide meaningful uncertainties in estimates. To better understand the results of N_2_O emission experiments and to evaluate the effectiveness of the sampling schemes used in mitigation experimentation, Bayesian methods are being developed to estimate cumulative N_2_O fluxes and to provide meaningful uncertainty estimates (Lehuger et al., 2009; Zhou et al., 2015; Cowan et al., 2017; Levy et al., 2017). Through better statistical handling of N_2_O measurement data, it is possible that uncertainties may be better understood and reduced in future experimentation.

To improve regional and national scale accounting for N_2_O emissions, it has been suggested that the IPCC Tier 1 method (De Klein, 2006), assuming a constant EF for all applied Nr is too simplistic, and that the development of a Tier 2 method which incorporates fertiliser type and environmental conditions should be considered (Skiba et al., 2012). However, it has also been recognized that due to the unpredictable nature of N_2_O emissions, large uncertainties in inventories may remain. To test these assumptions, coordinated experimentation is required at a national scale from multiple experimental sites.

This study aims to use a Bayesian method for estimating cumulative fluxes (Levy et al., 2017) to quantify the efficacy of a urease inhibitor in a series of experiments replicated across the UK. The experiments measure N_2_O EFs after application of Agrotain® (urease-inhibitor-treated urea, N-(n-butyl) thiophosphoric triamide, Koch, KS, USA) on intensively managed grassland silage crops at four sites in the UK, and compare this with the two most commonly used synthetic nitrogen fertilisers in the UK: ammonium nitrate (Nitram®, CF Fertilisers UK Ltd., Cheshire, UK) and urea. The results will then be used to analyse the feasibility of a Tier 2 EF method for regional and national N_2_O emission inventories.

## 2 Materials and methods

### 2.1 Field sites and experimental design

Four trials were conducted at intensively managed grassland sites in the UK, during the growing seasons in 2016 and 2017 (Table 1).

**Table 1:** Characteristics of the four field sites where fertiliser trials were carried out.

Site	Year	pH	Annual rainfall (mm)	Mean annual soil Temp. (°C)	Grass species	Previous management
EB	2016	6.02	793	9	*Lolium perenne* L.	Mostly sheep grazing
HF	2016	6.32	1250	11	*Lolium multiflorum* Lam.	Silage with winter grazing
NW	2016	5.77	1107	12	*Lolium perenne* L.	Silage with winter grazing
UJ	2017	6.10	780	10	*Lolium perenne* L.	Silage with winter grazing

The trials were carried out at Easter Bush farm estate (Midlothian, Scotland) (Drewer et al., 2016; Jones et al., 2017), Henfaes Research Station (Abergwyngregyn, Wales) (HF) (Shaw et al., 2016) and Rothamsted Research, (North Wyke, southwest England) (NW) (Rennie et al., 2017).

Two fields within the Easter Bush farm estate were used, referred to as Easter Bush (EB) and Upper Joiner (UJ) field sites. The EB field had historically been used to graze sheep (0.7 LSU ha^−1^) The UJ field had predominantly been used for silage harvest with occasional grazing during winter. The HF and NW sites were managed similarly, predominantly used for silage harvest with occasional grazing during winter months (Carswell et al., 2018).

Experimental plots were arranged at each site in strips of 2 m by 8 m (with a 0.5–2.0 m spacing between them), positioned randomly to mitigate bias resulting from spatial variability of soil properties. The plot layout varied in the 2017 UJ trial, for which plots were arranged in a square grid, each measuring 20 m by 20 m with no spacing between them. Applications of nitrogen fertilisers in the form of ammonium nitrate (AN), urea (Ur), and urea treated with the Agrotain® urease inhibitor (UI) were applied to the plots (via manual spreading) two or three times per site, each application was replicated on four plots with an additional four control plots to which no Nr fertiliser was applied (a total of 16 plots per fertiliser event; Table 2). Fertiliser applications were applied at 60, 70 or 90 kg N ha^−1^ based on typical farm practices at the respective sites.

**Table 2:** A summary of the nitrogen applications at the field sites. Equivalent quantities of total nitrogen were applied to four plots in the form of AN, U and UI for each event.

Site	Date Event 1	Total N applied (kg N ha^−1^)	Date Event 2	Total N applied (kg N ha^−1^)	Date Event 3	Total N applied (kg N ha^−1^)
EB	2016-03-11	70	2016-07-15	70	/	/
HF	2016-05-05	90	2016-06-13	90	2016-07-25	60
NW	2016-03-23	90	2016-05-19	90	2016-07-08	60
UJ	2017-05-25	70	2017-07-19	70	2017-09-15	70

### 2.2 N_2_O flux measurements

At all sites, measurements of N_2_O fluxes were taken using the static manual chamber approach. At the EB and UJ sites, chambers consisted of a cylindrical polyvinyl chloride (PVC) plastic pipe of 38 cm inner diameter (ID) and 22 cm height fitted with sealed lid and a flange at the base. The chambers were placed onto a plastic flanged collar that had been inserted several centimeters into the soil (on average 5 cm) to form a seal in the soil. A layer of draught sealant material held in place by four strong gripping clips formed an airtight seal between the chamber and the collar for the duration of the flux measurement. Chambers were closed for 60 min, during which four gas samples were collected via a syringe and a three-way tap fitted to the lid, at t = 0, 20, 40 and 60 min. Gas samples were stored in 20 ml glass vials, which were flushed with 100 ml of air from the syringe using a double needle. Samples were analysed using gas chromatography (7890B GC system fitted with an electron capture detector and 7697A Headspace Autosampler, Agilent Technologies, Santa Clara, California, United States). At the EB and UJ sites the manual static chamber measurements were carried out daily for two weeks after fertilisation, then every second day for a further two to four weeks, with measurements only made only on working days (Monday to Friday) between 09:00 and 15:00 GMT.

At the HF and NW sites, slots were cut into the soil and the chambers (50 × 50 × 30 cm) were inserted, so that 15–20 cm of the chamber height remained above the soil surface. On each sampling occasion, lids were placed on the chambers and remained in place for 40 min with three gas samples collected via syringe, at *t* = 0, 20 and 40 min. Gas samples were stored in pre-evacuated 20 ml glass vial, which were flushed with 50 ml of air from the syringe using a double needle. Samples were analysed for N_2_O concentration using a Perkin Elmer 580 Gas Chromatograph (linked to a TurboMatrix 110 Headspace Autosampler) (Carswell et al., 2018). At the HF and NW sites manual static chamber measurements were carried out following fertilisation at three times weekly for weeks one and two, twice weekly for weeks three and four, and once weekly thereafter. Measurements were made between 09:00 and 15:00 GMT.

Further measurements were made at the HF site using an automatic chamber approach (via an Isotopic N_2_O Analyser, Los Gatos Research Inc. San Jose, CA, USA). Chamber bases were inserted into the soil and the chambers (50 × 50 × 20 cm) attached to the bases at surface height to ensure an air tight seal. Closing and opening of the chambers was controlled by pneumatic actuators. The chambers closed for a 30 min measurement period (four times a day), during which the chamber-headspace was sampled via a sampling port at a rate of 1 l min^−1^ at a frequency of 0.1 Hz.

Fluxes were calculated as:

(1) $$F=\frac{\mathrm{dC}}{\mathrm{dt}}.\frac{\mathrm{\rho V}}{A}$$

where F is the gas flux from the soil (nmol m^−2^ s^−1^) d*C*/d*t* is the rate of change in the concentration in time in nmol mol^−1^ s^−1^ estimated by linear regression, *ρ* is the density of air in mol m^−3^, V is the volume of the chamber in m^3^ and A is the ground area enclosed by the chamber in m^2^.

### 2.3 Interpolation of N_2_O flux data

Cumulative fluxes over the experimental periods (30 days) were calculated using a Bayesian approach, taking into account the log-normal distribution of spatial samples and the lognormal peak-and-decay pattern in time (Levy et al., 2017). Based on the assumption that at a given time, N_2_O fluxes, F, are typically log-normally distributed in space, the probability density is given by:

(2) $$f\left(F\right)=1/\left(\sqrt{\left(2\pi \right)}\sigma_{\log}F\right)\exp\left(-\left({\left(\log\left(F\right)-\mu_{\log}\right)}^{2}/\left(2\sigma_{\log}^{2}\right)\right)\right)$$

where *μ*_log_ and *σ*_log_ are the location and scale parameters, equivalent to the mean and standard deviation of the log-transformed variate. The mean of the distribution is given by:

(3) $$\mu =\exp\left(\mu_{\log}+0.5\sigma_{\log}^{2}\right)$$

Following a fertilisation event, the time course of N_2_O flux is expected to rise to a peak, then decay exponentially, and this basic pattern is reproduced by all process-based models (i.e. Li et al., 1992; Del Grosso et al., 2006) and is also well described by the log-normal equation:

(4) $$\mu_{t}=1/\left(\sqrt{\left(2\pi \right)}\mathrm{kt}\right)\exp\left(-\left({\left(\log\left(t\right)-\Delta \right)}^{2}/\left(2k^{2}\right)\right)\right)\cdot N_{\mathrm{in}}\Omega$$

where *μ*_*t*_ is the spatial mean of the N_2_O flux at time t, *Δ* and k are analogues for the location and scale parameters, and with the additional term *N*_*in*_ is the fertiliser nitrogen input and *Ω* is the fraction of this which is emitted as N_2_O as *t* tends toward infinity. *Δ* can be interpreted as the natural logarithm of the delay between fertiliser application and peak flux; k is a decay rate term. So, at time t following fertilisation, the mean flux is given by Eq. (5), (6), at which time the N_2_O flux has a distribution

(5) $$F∼\ln N\left(\mu_{\log,t},\sigma_{\log}^{2}\right)$$

where

(6) $$\mu_{\log,t}=\log\left(\mu_{t}\right)-0.5\sigma_{\log}^{2}$$

The parameters *μ*, *μ*_log_ and *σ*_log_ were estimated using the Markov Chain Monte Carlo (MCMC) method with Gibbs sampling (Gelman, 2013). This was implemented using the freely available JAGS software (Plummer, 2016). The prior distribution for Ω was based on the data collated by Stehfest and Bouwman (2006). The prior distributions for Δ and k were based on the dynamics of the DNDC model (Li et al., 1992, as described in Levy et al., 2017). To obtain the cumulative flux at time t, we use the standard log-normal cumulative distribution function:

(7) $$F_{\mathrm{cum},t}=\Phi \left(\frac{\ln t-\Delta }{k}\right)N_{\mathrm{in}}\Omega$$

where *Φ* is the cumulative distribution function of the standard normal distribution.

To account for background fluxes (fluxes of N_2_O expected in the absence of any applied nitrogen), a cumulative background flux was estimated using the mean of the fluxes measured from the control plots during each event. This cumulative background estimate was then subtracted from the cumulative fluxes estimated for each treatment. The reported EFs in this study take background fluxes into account when reporting final values (Table 3, Table 4, Table 5).

**Table 3:** Cumulative fluxes estimated using linear and Bayesian interpolation methods over a 30 day period after ammonium nitrate fertiliser applications at the four field sites. Values presented represent 4 plots (*n* = 4) per event at each field site. Emission factors account for the effect of N application after the measured background flux has been deducted from cumulative totals.

Site	Event	Fertiliser applied	Background flux	Linear interpolation cumulative	Linear minus background	Bayes interpolation cumulative	95% C.I.		Bayes minus background	Linear EF	Bayes EF
		(kg N ha^−1^)	(kg N ha^−1^)	(kg N ha^−1^)	(kg N ha^−1^)	(kg N ha^−1^)	min	max	(kg N ha^−1^)	(%)	(%)
Ammonium nitrate											
EB	1	70	0.25	1.66	1.41	1.59	1.02	2.86	1.34	2.02	1.92
EB	2	70	0.19	0.31	0.11	0.45	0.32	0.68	0.25	0.16	0.36
HF	1	90	0.01	0.06	0.04	0.05	0.05	0.06	0.04	0.05	0.04
HF	2	90	0.04	0.14	0.10	0.15	0.13	0.16	0.10	0.11	0.12
HF	3	60	0.06	0.18	0.11	0.19	0.17	0.21	0.13	0.19	0.21
NW	1	90	0.23	0.88	0.65	1.65	0.96	3.50	1.43	0.73	1.59
NW	2	90	0.16	0.41	0.25	0.70	0.38	1.61	0.54	0.28	0.61
NW	3	60	0.07	0.10	0.03	0.20	0.14	0.34	0.14	0.06	0.23
UJ	1	70	0.92	1.50	0.59	1.39	0.97	2.26	0.48	0.84	0.68
UJ	2	70	0.51	0.43	−0.08	0.50	0.39	0.67	−0.01	−0.11	−0.01
UJ	3	70	0.93	1.66	0.73	1.53	1.08	2.34	0.60	1.05	0.85

**Table 4:** Cumulative fluxes estimated using linear and Bayesian interpolation methods over a 30 day period after all Urea (Ur) applications at the four field sites. Values presented represent 4 plots (n = 4) per event at each field site. Emission factors account for the effect of N application after the measured background flux has been negated from cumulative totals.

Site	Event	Fertiliser applied	Background flux	Linear Interpolation cumulative	Linear minus background	Bayes interpolation cumulative	95% C.I.		Bayes minus background	Linear EF	Bayes EF
		(kg N ha^−1^)	(kg N ha^−1^)	(kg N ha^−1^)	(kg N ha^−1^)	(kg N ha^−1^)	min	max	(kg N ha^−1^)	(%)	(%)
Urea											
EB	1	70	0.25	0.51	0.26	0.52	0.37	0.78	0.27	0.37	0.38
EB	2	70	0.19	0.23	0.03	0.30	0.24	0.40	0.11	0.05	0.15
HF	1	90	0.01	0.06	0.05	0.06	0.05	0.07	0.05	0.05	0.05
HF	2	90	0.04	0.28	0.24	0.25	0.22	0.28	0.21	0.26	0.23
HF	3	60	0.06	0.33	0.27	0.32	0.29	0.35	0.26	0.45	0.43
NW	1	90	0.23	0.32	0.09	0.63	0.36	1.43	0.40	0.10	0.45
NW	2	90	0.16	0.25	0.09	0.53	0.30	1.13	0.37	0.10	0.41
NW	3	60	0.07	0.11	0.04	0.18	0.11	0.37	0.12	0.07	0.19
UJ	1	70	0.92	0.89	−0.03	0.99	0.72	1.48	0.07	−0.04	0.10
UJ	2	70	0.51	0.81	0.31	1.06	0.64	2.10	0.55	0.44	0.79
UJ	3	70	0.93	1.08	0.15	0.97	0.77	1.27	0.04	0.22	0.05

**Table 5:** Cumulative fluxes estimated using linear and Bayesian interpolation methods over a 30 day period after all Urea with inhibitor (UI) applications at the four field sites. Values presented represent 4 plots (n = 4) per event at each field site. Emission factors account for the effect of N application after the measured background flux has been negated from cumulative totals.

Site	Event	Fertiliser applied	Background flux	Linear interpolation cumulative	Linear minus background	Bayes interpolation cumulative	95% C.I.		Bayes minus background	Linear EF	Bayes EF
		(kg N ha^−1^)	(kg N ha^−1^)	(kg N ha^−1^)	(kg N ha^−1^)	(kg N ha^−1^)	min	max	(kg N ha^−1^)	(%)	(%)
Urea & inhibitor											
EB	1	70	0.25	0.48	0.23	0.54	0.37	0.90	0.28	0.33	0.41
EB	2	70	0.19	0.23	0.04	0.29	0.23	0.40	0.10	0.06	0.14
HF	1	90	0.01	0.07	0.06	0.07	0.06	0.07	0.05	0.07	0.06
HF	2	90	0.04	0.19	0.15	0.18	0.16	0.19	0.14	0.17	0.15
HF	3	60	0.06	0.31	0.25	0.28	0.25	0.32	0.22	0.41	0.37
NW	1	90	0.23	0.10	−0.13	0.26	0.15	0.51	0.03	−0.14	0.03
NW	2	90	0.16	0.25	0.10	0.43	0.26	0.89	0.27	0.11	0.30
NW	3	60	0.07	0.07	0.01	0.16	0.09	0.33	0.09	0.01	0.15
UJ	1	70	0.92	1.13	0.22	1.33	0.87	2.46	0.41	0.31	0.58
UJ	2	70	0.51	0.49	−0.02	0.67	0.50	0.97	0.17	−0.03	0.24
UJ	3	70	0.93	1.26	0.33	1.22	0.89	1.83	0.29	0.46	0.41

## 3 Results

### 3.1 Measured N_2_O fluxes

**Fig. 1:**
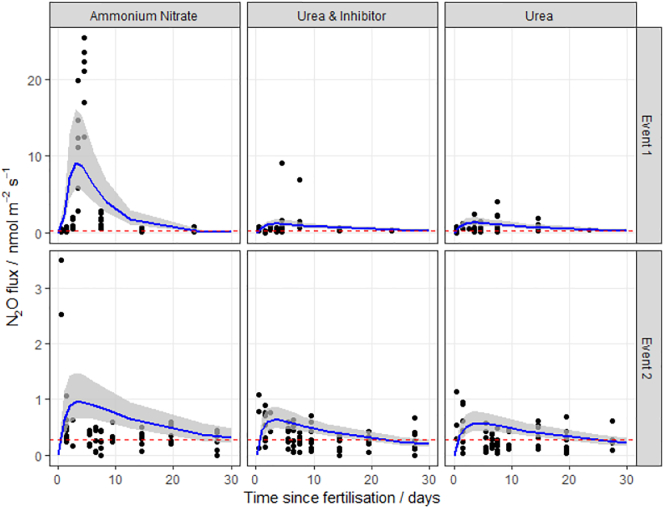
N_2_O fluxes following fertilisation with three different nitrogen forms at the Easter Bush field site (EB, Midlothian, Scotland) in 2016. The log-normal model was used to estimate cumulative N_2_O fluxes. The 95% credible intervals of the posterior predictions are shown as the shaded area. Mean background fluxes from control plots are included for each event (red dashed line). (For interpretation of the references to colour in this figure legend, the reader is referred to the web version of this article.)

**Fig. 2:**
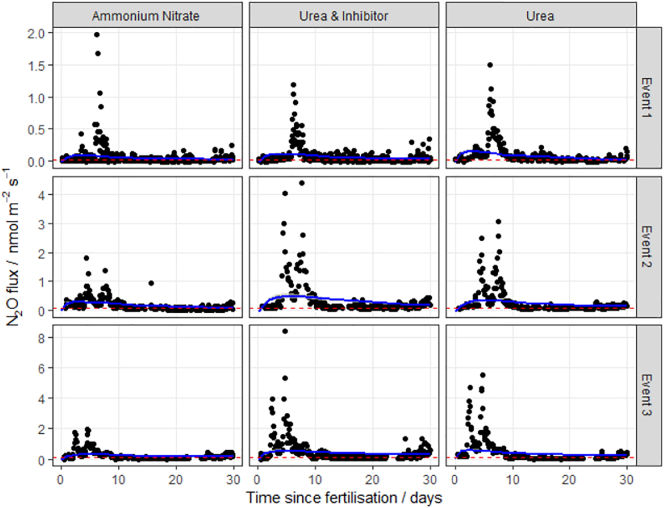
N_2_O fluxes following fertilisation with three different nitrogen forms at Henfaes Research Station (HF, Abergwyngregyn, Wales) in 2016. The log-normal model was used to estimate cumulative N_2_O fluxes. The 95% credible intervals of the posterior predictions are shown as the shaded area. Mean background fluxes from control plots are included for each event (red dashed line). (For interpretation of the references to colour in this figure legend, the reader is referred to the web version of this article.)

**Fig. 3:**
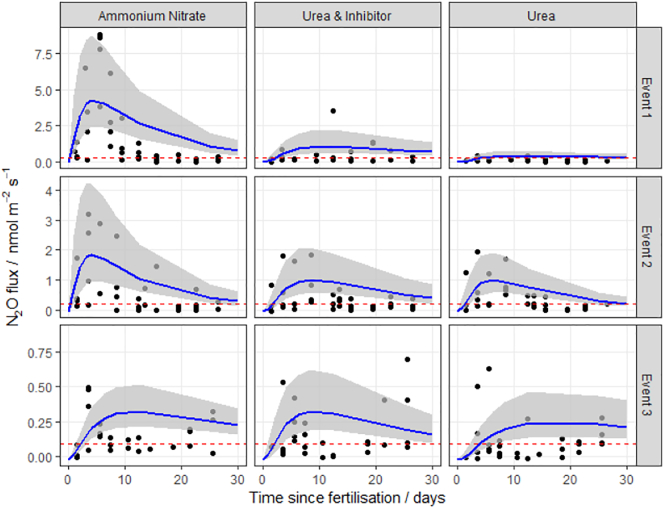
N_2_O fluxes following fertilisation with three different nitrogen forms at the Rothamsted Research site, (NW, North Wyke, southwest England) in 2016. The log-normal model was used to estimate cumulative N_2_O fluxes. The 95% credible intervals of the posterior predictions are shown as the shaded area. Mean background fluxes from control plots are included for each event (red dashed line). (For interpretation of the references to colour in this figure legend, the reader is referred to the web version of this article.)

**Fig. 4:**
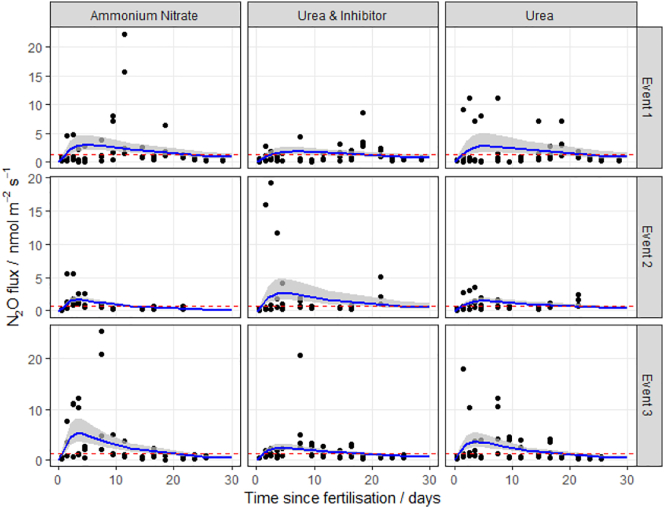
N_2_O fluxes following fertilisation with three different nitrogen forms at the Upper Joiner field site (UJ, Easter Bush, Midlothian, Scotland) in 2017. The log-normal model was used to estimate cumulative N_2_O fluxes. The 95% credible intervals of the posterior predictions are shown as the shaded area. Mean background fluxes from control plots are included for each event (red dashed line). (For interpretation of the references to colour in this figure legend, the reader is referred to the web version of this article.)

A log-normal spatial distribution of data was typically observed for each of the N application events where static chamber measurements were made, with individual chamber fluxes ranging between −0.02 and 25.4 nmol N_2_O m^−2^ s^−1^ (Fig. 1, Fig. 2, Fig. 3, Fig. 4). A variable, but significant increase in emissions of N_2_O in the days after the fertiliser event was broadly observed for all events, with a few exceptions. The time delay between N application and the observed increase in N_2_O flux varied by site and fertiliser type, although the vast majority of emissions appear to have occurred within the 30 day window. Fluxes from control plots varied across the sites and dates of application events; however, these emissions were relatively low with a mean value of 0.4 nmol N_2_O m^−2^ s^−1^.

The log-normal model generally fitted well to the measurements. The exception was at the HF site, where the pattern of emissions does not closely follow the log-normal pattern described in Eq.2 (Fig. 2), having very extreme but short-lived peaks. It is unclear why these data show the least correspondence with the log-normal pattern. Partly the fit is dominated by the large number of near-zero fluxes in the tail of the distribution. Partly it may be due to the small number of spatial samples used in the autochamber system at this site. Each of these has a different timing of the peak, and the ensemble mean does not closely follow the log-normal pattern. The use of other meta-models needs to be explored in this case.

### 3.2 Cumulative fluxes and emission factors

Cumulative fluxes calculated using the linear and Bayesian methods were broadly comparable across the different sites. The direct comparison of the two interpolation methods shows that in this study the Bayesian method predicted slightly larger fluxes than the linear method (slope = 1.04) and there is some disagreement in larger EF estimates (R^2^ = 0.79), but overall the comparison was good. Taking background emissions into account, EFs ranged from slightly negative values (−0.01%, where treatment plots emitted less N_2_O than control plots) to a maximum of 1.92% of the N applied.

**Fig. 5:**
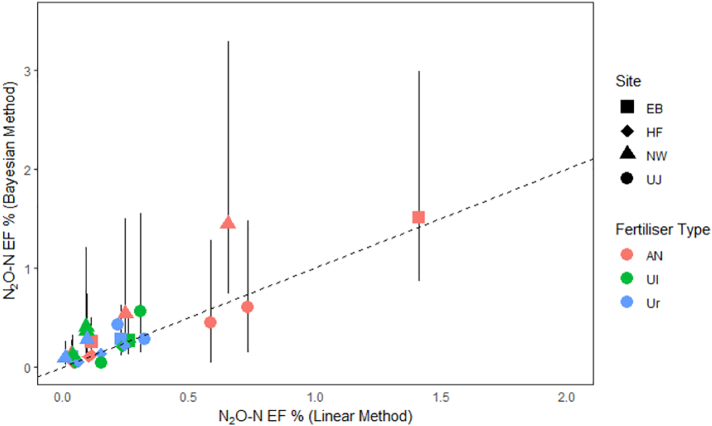
Comparison of the Emission factors estimated using the trapezoidal (linear) and log-normal Bayesian integration methods of all fertiliser applications at four sites in the UK.

A large degree of relative variability was observed between EFs reported for the different fertiliser types within the same field site. On occasions, when the measurements were particularly variable, uncertainties in cumulative fluxes estimated using the Bayesian method were greater than 1% of the applied N. In these cases neither method was able to determine accurate cumulative estimates based on the available measurement data; this is represented in the large uncertainty value reported by the Bayesian method (Fig. 5).

**Fig. 6:**
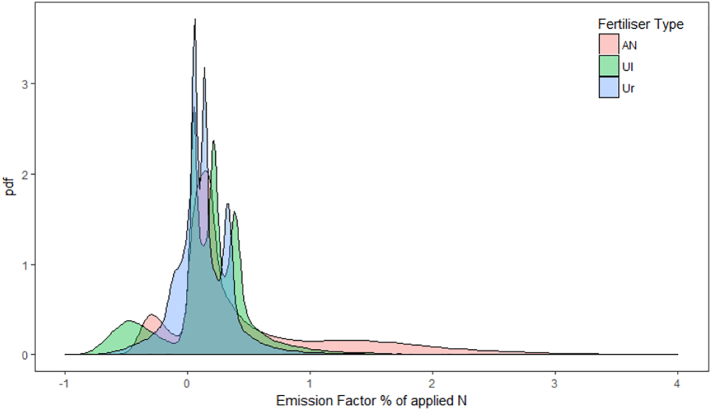
The probability distribution function (pdf) of N_2_O emission factors of applied nitrogen estimated using the log-normal Bayesian interpolation method for 11 fertiliser applications at four sites in the UK.

By combining the cumulative fluxes calculated by the MCMC chains when using the log-normal Bayesian method, we can observe the posterior distributions of the EFs predicted for each treatment across all experiments (Fig. 6). The overlap of the distributions highlights the similarity in emissions observed between the treatments; although the shape of the distribution is distinctly different between the urea and AN treatments with a higher probability of observing EFs above 1% for AN.

## 4 Discussion

Mean EFs estimated using the Bayesian interpolation method were 0.60 ± 0.63, 0.29 ± 0.22 and 0.26 ± 0.17% for the AN, Ur and UI treatments, respectively. These observations are within the range of results reported in similar experiments for which N_2_O EFs for applications of synthetic N fertilisers such as AN and Ur vary in the region of 0 to 3% of applied N (Dobbie et al., 1999; IPCC, 2014; Akiyama et al., 2006; Stehfest and Bouwman, 2006), although values are well below the 1% IPCC default value. The AN treatment occasionally exceeded the 1% default EF value, although most of the individual EFs for all treatments remained below this value in this study (median EF = 0.24%).

Emissions associated with either urea treatment applications occasionally surpassed the emissions from the AN treatments, but EFs for both the urea treatments consistently remained below the IPCC 1% estimate (after background fluxes were taken into account), not breaching the 0.5% mark in any experiment (although 95% C.I.s reached values greater than 1.5% on occasion). Based on the posterior distribution provided by the Bayesian method, EFs associated with AN treatments were found to be larger than those of the urea treatments 55% of the time and that the EFs associated with UI were larger than Ur 60% of the time; however, the magnitude of these differences was inconsistent and not statistically significant.

Our results agree with previous studies that AN fertilisers can emit more N_2_O than equivalent applications of urea (Harty et al., 2016); however, this study highlights that there is a large degree of variability between each individual event and that on occasion, urea application can emit more than AN fertilisers, as has been observed in other studies (Bouwman, 1996; Smith et al., 1997; Bell et al., 2015). Our study suggests that generally there is no significant increase in the production of N_2_O when the Agrotain® urease inhibitor is applied to urea fertiliser; however, this study highlights that due to the unpredictable nature of N_2_O fluxes and the methods typically used to measure them, individual experiments are likely to see a wide range of outcomes even if there were no real treatment differences. This may explain the wide range of observations in experiments investigating N_2_O emissions after using inhibitors with fertiliser applications (Lam et al., 2016; Singh et al., 2013; Rose et al., 2017; Ruser and Schulz, 2015).

The reported fluxes and EFs in this study follow a log-normal distribution in both space and time, as is regularly observed in measurement data (Stehfest and Bouwman, 2006). Clear differences were difficult to establish between treatments, which is typical for data that follow a log-normal distribution, as demonstrated in Fig. 6. This observation highlights two major issues with fertiliser comparison experiments. Firstly, based on the variability in these observations, a very large number of replicates is needed when assessing treatments with small effect sizes. This presents problems when resources to carry out such large experiments is limited. The second issue is that the log-normal distribution of the data complicates the analysis considerably. Simply log-transforming the data does not suffice, because we are interested in properties of the data in untransformed space, such as the mean. The Bayesian method applied here provides a means of tackling this problem.

The wide variation in emissions after fertilisation events is often attributed to multiple environmental variables (such as temperature, oxygen availability, soil type, soil moisture, plant residue, soil carbon content, pH). However, it is very difficult to accurately predict the microbial interactions which affect N_2_O production from the information that is available in most experiments (Bouwman, 1996). Correlations between cumulative fluxes and environmental variables are statistically weak and inconsistent between the events at different sites. What is apparent is that the EF for a given fertilisation event is very unpredictable. The same fertiliser type applied at the same site under similar conditions can produce quite different emissions on different occasions, for reasons we do not always understand, and which cannot always be predicted from the principles in process-based models (Butterbach-Bahl et al., 2013).

Our study highlights several points of interest that should be taken into account when Tier 2 EFs are being considered. The first point is that different fertiliser types have a wide range of EFs that may not be predicted using the obvious, easily measured environmental variables, such as rainfall or soil mineral N concentrations. In this study, none of the measured environmental or soil variables were able to explain the variation in emission factors. The second point is that microbial produced emissions for each individual event are influenced by a wide number of factors and vary with a log-normal exponential behavior, potentially emitting an unpredictably large quantity of N_2_O over several hours that could be greater than cumulative emissions for several weeks afterwards, or alternatively, nothing at all under what appears to be similar conditions (as observed in Skiba et al., 2013, and Levy et al., 2017). As such, the application of usual meteorological and environmental conditions used to predict N_2_O emissions at a Tier 2 level is unlikely to reduce uncertainties at the regional or national scale any more than the application of a Tier 1 inventory, as predicted in Skiba et al. (2012).

What is noticeable in our study, is that the EFs of different fertiliser types have different probability density functions (pdfs), as demonstrated by the contrast of AN and urea treatments (Fig. 6). With a large number of experiments across the region of interest, these pdfs will become more defined and may better represent the emissions expected from a particular event than the default 1% EF suggested by the IPCC report. The exact number of experiments required to accurately predict a regional pdf is unknown, because of the difficulty of measuring the exact soil conditions within the soil microsites were N_2_O is produced.

A comparison of the Bayesian and trapezoidal linear interpolation methods show that they did not differ systematically, but the former provides rigorous uncertainties when a limited number of data points is available. In this respect, the Bayesian method has been a success, as large uncertainties are provided when data varies significantly in time and space, something which was not possible to assess when using traditional trapezoidal linear interpolation method. However, care should be taken when using the Bayesian method when flux measurements are more temporally defined (i.e. auto-chambers and eddy covariance), as the log-normal fit becomes too constrained and uncertainty estimates are no longer valid.

## 5 Conclusions

A Bayesian approach was used to calculate EFs and their associated uncertainties from the application of nitrogen fertilisers in the form of ammonium nitrate (AN), urea (Ur) and urea treated with Agrotain® urease inhibitor (UI) at four grassland sites in the UK. The study reports that the EFs observed after 11 separate events followed a variable log-normal distribution, with mean reported values of 0.60 ± 0.63, 0.29 ± 0.22 and 0.26 ± 0.17% of applied N emitted as N_2_O for the AN, Ur and UI treatments, respectively. The study found that EFs associated with AN were more likely to be larger than those of the urea-based treatments, but there was no significant difference in overall emissions of N_2_O between the Ur and the UI treatments. The Bayesian method used in this study successfully provided uncertainty values in cumulative fluxes of N_2_O that traditional trapezoidal linear interpolation methods could not. In its current form, the method is limited to cases where the emissions show a peak and decline following fertilisation, as expected from first principles.
